# GenBank and PubMed: How connected are they?

**DOI:** 10.1186/1756-0500-2-101

**Published:** 2009-06-09

**Authors:** Holly Miller, Catherine N Norton, Indra Neil Sarkar

**Affiliations:** 1 MBLWHOI Library, Marine Biological Laboratory, 7 MBL Street, Woods Hole, MA 02543, USA; 2 Current address: Center for Clinical and Translational Science, University of Vermont, Burlington, VT 05401, USA

## Abstract

**Background:**

GenBank(R) is a public repository of all publicly available molecular sequence data from a range of sources. In addition to relevant metadata (e.g., sequence description, source organism and taxonomy), publication information is recorded in the GenBank data file. The identification of literature associated with a given molecular sequence may be an essential first step in developing research hypotheses. Although many of the publications associated with GenBank records may not be linked into or part of complementary literature databases (e.g., PubMed), GenBank records associated with literature indexed in Medline are identifiable as they contain PubMed identifiers (PMIDs).

**Results:**

Here we show that an analysis of 87,116,501 GenBank sequence files reveals that 42% are associated with a publication or patent. Of these, 71% are associated with PMIDs, and can therefore be linked to a citation record in the PubMed database. The remaining (29%) of publication-associated GenBank entries either do not have PMIDs or cite a publication that is not currently indexed by PubMed. We also identify the journal titles that are linked through citations in the GenBank files to the largest number of sequences.

**Conclusion:**

Our analysis suggests that GenBank contains molecular sequences from a range of disciplines beyond biomedicine, the initial scope of PubMed. The findings thus suggest opportunities to develop mechanisms for integrating biological knowledge beyond the biomedical field.

## Discussion

### Overview of GenBank

The US Congress established National Center for Biotechnology Information (NCBI) in 1988 to develop bioinformatics approaches to support the progress of biomedical research. A major component of NCBI's mission is to provide access to a variety of databases and software for the scientific and medical communities. GenBank [1], an archive of all publically available primary sequence data, is one of these databases. Sequence data are submitted to GenBank from individual scientists and from large genome sequencing centers. GenBank, the European Molecular Biology Laboratory Nucleotide Sequence Database (EMBL) in Europe, and the DNA Databank of Japan (DDBJ) together form the International Nucleotide Sequence Database Collaboration (INSDC). The INSDC archives and makes publically available more than 80 million individual molecular sequences including mRNA sequences, genomic survey sequences and ribosomal RNA gene clusters [1]. Data is exchanged daily among the INSDC partners (GenBank, EMBL, and DDBJ) to maintain consistency and completeness of molecular sequence data contributed and used by the scientific community.

### Accuracy and completeness of GenBank

In addition to the actual molecular sequence, each GenBank entry includes a set of associated metadata that provide information about each sequence. These metadata elements include a description of the sequence, the scientific name of the organism, bibliographic information citing relevant publications, taxonomy of the source organism, and a features table providing information about coding regions, translated protein sequence, repeat sequences and many other relevant characteristics of the sequence. The importance and accuracy of GenBank annotations has been the topic of recent discussions [2]. Community-based curation of GenBank annotations has been suggested as a tractable means to keep pace with high-throughput genome sequencing initiatives [3,4]. Currently, NCBI performs some quality control, but does not curate the data; only the original submitter can make modifications to a molecular sequence record including the annotation in GenBank [3].

Many, if not all, biology and biomedical journals require that authors submit manuscript associated molecular sequence data to public repositories such as GenBank. There have been recent studies exploring author compliance with such requirements – Noor *et al. *report that 3–20% of biomedical journal articles do not have requisite GenBank accession numbers in accordance with journal policies [5]. In many cases, the requisite molecular sequence data is not available even six months after publication [5]. However, many molecular sequences are submitted by generous scientist(s) who have no plans to formally publish the research and in this case no citation information would be in the GenBank record.

### Link between GenBank sequences and publications

The identification of literature associated with a given molecular sequence may be an essential first step in developing research hypotheses. Thus, the connection of GenBank records to peer-reviewed, published literature is an essential component of contemporary biomedical research. Many of the publications associated with GenBank records may not be linked into or part of complementary literature databases (e.g., PubMed). GenBank records associated with literature indexed in Medline are identifiable as they contain PubMed identifiers (PMIDs). From the perspective of molecular sequence data, it can be essential to have access to the associated publication to address quality or methodology inquiries [6]. For example, quality issues are of paramount importance when combining molecular sequence data for identification, population and evolutionary studies [6-8]. Having an associated publication linked to a given GenBank record enables one to confirm the methodology used to acquire the molecular sequences (e.g., specimen handling or PCR primers used) in addition to the context under which the sequence was studied (e.g., field collection or laboratory extraction). A link to published literature can also be a means to explore data behind proposed gene function or identify other related experiments (e.g., gene expression studies).

To analyze the connections between GenBank and published literature, a full GenBank archive (release 164) was downloaded in flat-file format from the NCBI at the National Library of Medicine in March 2008. The downloaded flat files were then parsed to extract 70 metadata types associated with each GenBank record. Annotation values for each of the 70 metadata types were then loaded into a MySQL database. Citation information was gathered from the JOURNAL GenBank Feature Table field; PubMed identifiers were extracted from the PMID GenBank Feature Table field. A citations table was thus created that contained: GenBank Identifier, full citation, journal name, and PMID (when available) and a series of MySQL queries were then crafted to calculate the statistics reported.

**Figure 1:**
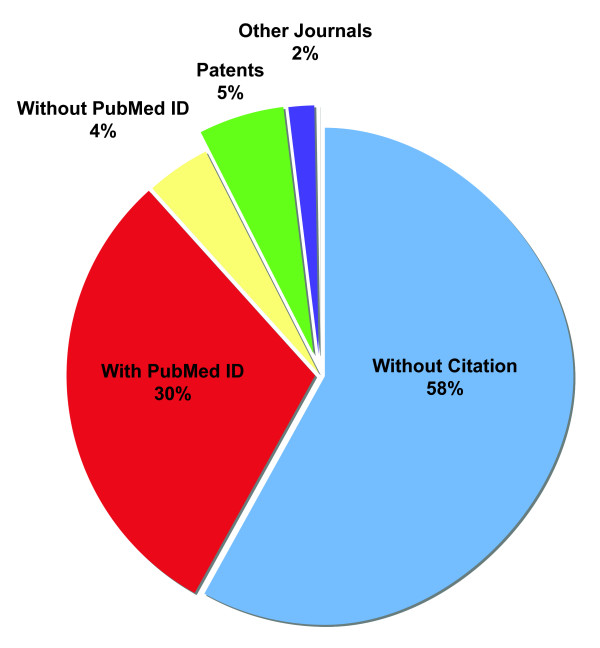
**Distribution publication type information associated with Genbank sequences**. Pie-chart showing the percentage of Genbank records that have: no associated citation information (blue; 50,480,022 records), a PubMed ID indicating the publication's abstract is available from NCBI's PubMed database (red; 26417760 records), citation information corresponding to PubMed indexed publications but no PubMed ID (yellow; 3,641,494 records), a patent citation (green; 4,830,186 records), or citation information corresponding to a journal not indexed in the PubMed database (purple; 1,747,039 records).

An examination of 87,116,501 GenBank records indicates that 42% are associated with some literature citation information (Figure 1). Most of these records (71%) are associated with a PubMed Identifier (PMID). Of the records that are associated with a citation but not indexed by PubMed (as inferred by the lack of a PMID – 29%), we found that 73% are associated with patent publications and 26% with journals that are not indexed by PubMed. Interestingly, a number of GenBank records that are associated with publications that are indexed in PubMed lack PMIDs (9% of all citations). This may be reflective of dependence on submitters to update their GenBank records as a PMID becomes available. Recently, this topic has been raised in a letter to Nature that urges submitters to all three databases to check their records and update the information [9]. Information for submitters on the EMBL website  encourages researchers to include the accession number of the relevant sequences in the publications and to let EMBL staff know where the data they have submitted have been published. As only the submitting group can authorize changes and updates to sequence records, the ultimate responsibility for the quality of sequence data the public has access to is in the hands of the scientists who submit the data. An added benefit of researchers providing accession numbers in articles is that this information can be extremely useful to readers of the paper who would like to know which exact gene sequence the authors of the paper have been studying. Furthermore it would allow bioinformaticians to analyze the number of articles that report data related to individual GenBank sequences, something very difficult to at present.

### Journals linked with largest number of GenBank sequences

Citation data associated with GenBank records enables one to identify the frequency at which particular journals are linked to molecular data deposited in GenBank. For journals indexed in PubMed, *PLoS Biology *has the highest number of GenBank citations; over 7% of the sequences in GenBank cite *PLoS Biology *and nearly 18% of the sequences that contain citations (Table 1). *Genome Research *and *The Proceedings of the National Academy of Sciences of the United States of America *(*PNAS*) are cited in nearly 14% and 8% of GenBank entries with sequences, respectively. In the case of *PLoS Biology*, however, the number of sequences published is significantly affected by three articles pertaining to recent metagenomic studies from J. Craig Venter Institute's Global Ocean Sampling expedition, which resulted in the deposition of millions of sequences from oceanic microbes collected during the Sorcerer II expedition [10-12]. In Table 1, the numbers in parentheses in the columns "Average number of sequences per article" and "Number of sequences" are the values without including sequences from these three articles.

**Table 1:** Journals Indexed in PubMed Linked to Most Sequences (Including Genome Sequencing Projects)

	**Citations in GenBank Sequence Records**			
	
**Journal Name**	**Percent of sequences associated with a citation**	**Ave #seqs/article**	**Number of distinct journal articles**	**Number of sequences**
PLOS Biology	17.7	60199 (32517)*	108 (105)	6501501(3414295)
Genome Research	13.9	10091	505	5095937
The Proceedings of the National Academy of Sciences	7.6	325	8564	2779264
Science	5.4	1299	1532	1989825
Nature	4.6	703	2384	1675206
Genome Biology	4.0	19737	74	1460541
Plant Physiology	2.5	430	2173	935009
BMC Genomics	2.0	2975	247	734823
Plant Molecular Biology	1.7	235	2695	633337
Nature Genetics	1.3	694	710	492838

* Values in parentheses are generated after the exclusion of the metagenomic papers from the Global Ocean Sampling expedition ([10-12])

Citation data extracted from the GenBank records was used to determine how many sequences are associated with each journal. This table lists the top ten journals indexed in PubMed as defined by the number of GenBank sequences that cite an article in that journal. The percentage of sequences associated with the journal is calculated from the total number of GenBank entries that have some citation information. As described in the discussion, three article *in PLoS Biology *resulted in the more than 3 million sequences being deposited in GenBank. These adjusted calculated values based on omitting theses sequences are shown in parentheses.

**Figure 2:**
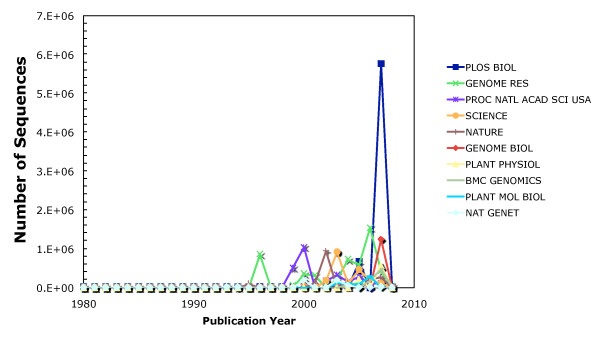
**Number of sequences associated with the "Top 10 Journals" by year**. Graph showing the number of sequences associated with articles published in the journals listed in Table 1 per year.

Considering the number of distinct articles that are linked to GenBank from each journal, *PNAS *has the highest number of articles linked to GenBank sequence data, followed by *Nature *and *Plant Physiology *(Table 1). Figure 2 shows the number of sequences linked to journals as a function of publication year. As expected, the number of sequences published has increased over time.

Many of the articles that are linked to large numbers of sequences are the result of high-throughput sequencing, therefore the number of sequences linked to that journal reflect that. This can been seen in the Ave # of seqs/article column of Tables 1 and 3. For example, *Genome Research *as an average of more 10,000 sequences linked per article and *Genome Research *has nearly 20,000 (Table 1). Therefore the data was reanalyzed eliminating articles that are cited by 15 or more sequences (Table 2). With the extremely sequence rich articles are removed from the analysis, the *Journal of Biological Chemistry *is now linked to the highest number of sequences with *PNAS *have slightly fewer sequence links.

**Table 2:** Journals Indexed in PubMed Linked to Most Sequences

**Journal Name**	**Number of sequences**
Journal of Biological Chemistry	21873
The Proceedings of the National Academy of	17388
Sciences	
Gene	14487
Nucleic Acids Research	11408
Journal of Bacteriology	10633
Applied Environmental Microbiology	8512
Genomics	8275
Biochem. Biophys. Research Communications	7088
Journal of Virology	6051
Int Journal of Systematic Evol Microbiol	5502

Citation data extracted from the GenBank records was used to determine how many sequences are associated with each journal. The data was filtered such that only articles with 14 or fewer sequences attributed to them were considered in order to eliminate article that result from large genome sequencing projects. This table lists the top ten journals indexed in PubMed as defined by the number of GenBank sequences that cite an article in that journal.

**Table 3:** Journals Not Indexed in PubMed Linked to Most Sequences (Including Genome Sequencing Projects)

	**Citations in GenBank Sequence Records**			
	
**Journal Name**	**Percent of sequences associated with a citation**	**Ave #seqs/arti cle**	**Number of distinct journal articles**	**Number**
Genetics and Molecular Biology	0.65	10331	23	237603
Breeding Science	0.21	4366	18	78594
Molecular Plant	0.14	525	100	52510
Pathology Journal of Phycology	0.10	103	368	37808
Molecular Ecology Resources (Formerly Molecular Ecology Notes)	0.08	19	1556	28898
Systematic Botany	0.07	73	359	26374
Integrative and Comparative Biology	0.06	1776	13	23092
Plant Biotechnology	0.05	302	62	18753
Phycologia Plant Molecular	0.04	243	77	18738
Biology Reporter	0.04	702	20	14036

Citation data extracted from the GenBank records was used to determine how many sequences are associated with each journal. This table lists the top ten journals not indexed in PubMed as defined by the number of GenBank sequences that cite an article in that journal. The percentage of sequences associated with the journal is calculated from the total number of GenBank entries that have some citation information.

Of the journals not indexed in PubMed, *Genetics and Molecular Biology *is cited in 237,603 GenBank sequences or 0.65% of the sequences are associated with a citation. The sequences associated with those journals not indexed by PubMed represent a significant source of sequence data (over 1.7 million sequence records). Table 3 shows the journals that have the highest number of GenBank sequence citations that are from journals not indexed in PubMed. The top journal in this ranked list is *Genetics and Molecular Biology *(ISSN 1415–4757; formerly *Brazilian Journal of Genetics*) is published by Sociedade Brasileira de Genética. None of the GenBank records from this journal have direct Web links to any of the articles associated with the over 230,000 sequences to the published articles, even though the journal abstract is electronically accessible through indices like ISI Web of Science and Biological Abstracts.

### Links to cited publication in GenBank record

GenBank, a resource maintained at the US National Library of Medicine (NLM), currently provides Web links only to those journals that are indexed in PubMed (which is also maintained at the NLM). The European Molecular Biology Laboratory (EMBL), which shares (along with the DNA Databank of Japan) molecular sequence records as part of the International Nucleotide Sequence Database Collaboration, uses Digital Object Identifiers (DOI^®^; ) for citations that do not have PMIDs. EMBL also includes links to other complementary citation databases, like the AGRICOLA database . For example, a sampling of GenBank sequence records associated with publications in *Molecular Plant Pathology *were manually compared to equivalent records in the EMBL database and found to contain direct links to the cited article at the publisher site (via the DOI) and to the abstract in AGRICOLA. However, further exploration of EMBL records suggests that incorporation of DOIs is not uniformly applied. For example, we found that articles from *Molecular Ecology Resources *and *Systematic Botany *are not associated with DOIs, even though both journals make use of DOIs.

### GenBank records that lack PMIDs

A closer examination of GenBank records that lack PMIDs but are associated with journals that have PMIDs reveals some interesting trends. Looking specifically at four journals that had a large proportion of GenBank citations without PMIDs suggests that there are significant gulfs in electronic linkages between molecular data and corresponding literature. *PLoS Biology*, for example, is cited in ~6.5 million GenBank entries. However, more than 40% of these records are missing PMIDs (which we identified through manual searches in PubMed). There were some instances where a PMID was available in the corresponding EMBL record, indicating that there may be some important parts of the sequence record are not exchanged as part of the INSDC relationship. Of note, nearly 70% of these sequences are associated with a single article [9]; overall, only 13 distinct articles are cited in these 3.8 million entries. This suggests that authors of manuscripts associated with molecular sequence data need to be diligent in updating their submissions such that the community may benefit from the electronic linking of molecular sequence to relevant literature [9]. In other instances, there was no clear pattern of GenBank entries that had citation information without PMIDs. For example, GenBank entries associated with *Molecular Ecology *are missing PMIDs for nearly 20% of the citations. On checking a number of these entries manually, we discovered that several of these entries have PMIDs listed in the corresponding EMBL record.

For a small portion (606 records), we encountered cases where PMIDs were the only information associated with the GenBank record (i.e., no additional citation information was available in the GenBank Record). Examination of the citation information from PubMed using the PMIDs shows that nearly half of these missing citations were published in *Plant Biology *(Stuttgart, Germany; ISSN 1435–8603). There is no apparent explanation as to why the full citations are missing from the GenBank records.

### Linking molecular sequences and scientific literature

The continually increasing size of molecular sequence and other scientific databases underscores the importance of linking information across relevant resources. There is currently much molecular and literature data available in electronic resources that can already be connected using existing technologies. GenBank and PubMed are two key resources that are well linked (although with some gaps). Still, a large portion of citation data in GenBank (~30% of GenBank records associated with some citation information) is not indexed as part of PubMed. A number of sequences are not associated with PMIDs and therefore not readily linkable to other spheres of knowledge (i.e., contained in relevant literature). Where PMIDs are not available, DOIs may be an alternative solution. For example, the presence of DOIs in the EMBL database enables access to literature beyond the scope of PubMed (e.g., biodiversity or ecology journals). Discussion with members of the NCBI staff indicates that DOIs are available in the raw GenBank record (ASN.1 format); however, they are not presented in the standard GenBank Website or through the flat-file download of GenBank records (Pers. Comm. – Scott Federhen & Mark Cavanaugh/NCBI/April 4, 2008).

It is an illuminating exercise to compare the presentation of the same sequence in all three databases. For example Accession BC002701, Homo sapiens ATM interactor, mRNA. The GenBank record at NCBI  has the PubMed id linked to the abstract for the cited *PNAS *article. The EMBL record  has a DOI and a PubMed, both of which are linked to the web resources to allow easy access to the article abstract and full text, as this article in PNAS is available for free. The DDBJ record displays the PubMed id but it is not linked to the abstract in PubMed.

An additional problem in connecting relevant literature to gene sequence data arises when considering whole genome sequencing projects. It is common for the large number of sequences derived from such projects to be linked to a single article that has little or no information related to the particular sequence. When sequences that are linked to 'sequence-rich' articles and are therefore probably part of large sequencing projects are excluded, only 6% of GenBank sequences are linked to articles that we postulate will offer pertinent and extensive information about the sequence.

## Conclusion

As we aspire for a truly connected universe of knowledge, where machines are able to communicate and even infer new correlations, it will be increasingly essential to have accurate and complete linkages across relevant resources. GenBank, along with its INSDC partners (EMBL & DDBJ), are not only archival stores of molecular sequence data but can also be considered starting points for future studies. As GenBank continues to grow beyond a predominantly biomedical resource and incorporated into non-biomedical research inquiries, it will be necessary to consider means to link additional electronic indices associated with non-biomedical biological literature.

## Competing interests

The authors declare that they have no competing interests.

## Authors' contributions

HM, CNN and INS analyzed the data and wrote the paper. INS wrote the Ruby scripts to download and parse the GenBank data into a MySQL database.

## References

[B1] Benson DA, Karsch-Mizrachi I, Lipman DJ, Ostell J, Sayers EW (2009). GenBank. Nucleic Acids Res.

[B2] Bidartondo MI (2008). Preserving accuracy in GenBank. Science.

[B3] Pennisi E (2008). DNA data. Proposal to 'Wikify' GenBank meets stiff resistance. Science.

[B4] Salzberg SL (2007). Genome re-annotation: a wiki solution?. Genome Biol.

[B5] Noor MA, Zimmerman KJ, Teeter KC (2006). Data sharing: how much doesn't get submitted to GenBank?. PLoS Biol.

[B6] Harris D (2003). Can You Bank on GenBank. Trends Ecol Evol.

[B7] Ryberg M, Nilsson RH, Kristiansson E, Topel M, Jacobsson S, Larsson E (2008). Mining metadata from unidentified ITS sequences in GenBank: a case study in Inocybe (Basidiomycota). BMC Evol Biol.

[B8] Valkiunas G, Atkinson CT, Bensch S, Sehgal RN, Ricklefs RE (2008). Parasite misidentifications in GenBank: how to minimize their number?. Trends Parasitol.

[B9] Ouellette F (2001). Users must help to keep public databases correct. Nature.

[B10] Kannan N, Taylor SS, Zhai Y, Venter JC, Manning G (2007). Structural and functional diversity of the microbial kinome. PLoS Biol.

[B11] Rusch DB, Halpern AL, Sutton G, Heidelberg KB, Williamson S, Yooseph S, Wu D, Eisen JA, Hoffman JM, Remington K (2007). The Sorcerer II Global Ocean Sampling expedition: northwest Atlantic through eastern tropical Pacific. PLoS Biol.

[B12] Yooseph S, Sutton G, Rusch DB, Halpern AL, Williamson SJ, Remington K, Eisen JA, Heidelberg KB, Manning G, Li W (2007). The Sorcerer II Global Ocean Sampling expedition: expanding the universe of protein families. PLoS Biol.

